# The molecular etiology of deafness and auditory performance in the postlingually deafened cochlear implantees

**DOI:** 10.1038/s41598-020-62647-y

**Published:** 2020-04-01

**Authors:** Sang-Yeon Lee, Ye Ji Shim, Jin-Hee Han, Jae-Jin Song, Ja-Won Koo, Seung Ha Oh, Seungmin Lee, Doo-Yi Oh, Byung Yoon Choi

**Affiliations:** 1 Department of Otorhinolaryngology-Head and Neck Surgery, Seoul National University Bundang Hospital, Seongnam, South Korea; 2 Department of Otorhinolaryngology-Head and Neck Surgery, Healthcare System Gangnam Center, Seoul National University Hospital, Seoul, South Korea; 3 Department of Otorhinolaryngology-Head and Neck Surgery, Seoul National University Hospital, Seoul, South Korea

**Keywords:** Health care, Medical research, Molecular medicine

## Abstract

Recent advances in molecular genetic testing (MGT) have improved identification of genetic aetiology of candidates for cochlear implantation (CI). However, whether genetic information increases CI outcome predictability in post-lingual deafness remains unclear. Therefore, we evaluated the outcomes of CI with respect to genetic aetiology and clinical predictors by comparing the data of study subjects; those with an identified genetic aetiology (GD group), and those without identifiable variants (GUD group). First, we identified the genetic aetiology in 21 of 40 subjects and also observed genetic etiologic heterogeneity. The GD group demonstrated significantly greater improvement in speech perception scores over a 1-year period than did the GUD group. Further, inverse correlation between deafness duration and the 1-year improvement in speech perception scores was tighter in the GD group than in the GUD group. The weak correlation between deafness duration and CI outcomes in the GUD group might suggest the pathophysiology underlying GUD already significantly involves the cortex, leading to lesser sensitivity to further cortex issues such as deafness duration. Under our MGT protocol, the correlation between deafness duration and CI outcomes were found to rely on the presence of identifiable genetic aetiology, strongly advocating early CI in individual with proven genetic aetiologies.

## Introduction

Cochlear implantation (CI), which is the surgical insertion of a neuroprosthetic electrode that bypasses the cochlear hair cells and directs sound sensations to the spiral ganglion neurons (SGN), has proven to be effective in auditory rehabilitation practices, such as speech performance, reading skills, and cognitive development of subjects with severe-to-profound sensorineural hearing loss (SNHL)^1^. The CI outcomes, however, appear to vary according to individual clinical characteristics, including the duration of deafness, age during CI operation, duration of hearing aids use, residual hearing, cochlear nerve integrity, and neurocognitive function^2–6^. Moreover, two previous studies conducted with data from 2251 adult patients implanted reported that only 10.5% and 22% of the variance in postoperative speech perception were influenced by 9 known clinical factors^7,8^. Although a recent advanced machine learning model has enabled clinicians to predict the potential benefits from CI by using various clinical variables^9^, the predictive ability of the model is limited due to considerable variations across individuals, especially those with longer duration of deafness (>10 years), suggesting that individualized physiological factors reflect the pathophysiology of SNHL.

Recent advancements in molecular genetic testing (MGT) have allowed for increase in the identification of causative genetic aetiology of SNHL in CI implantees^10–12^. Previous studies suggested that some genetic variants are closely associated with auditory performance after CI^11,13,14^. For example, individuals carrying specific variants in the *MYO15A, TECTA, and ACTG1* genes demonstrated better CI outcomes, whereas those with pathogenic mutations in *PCDH15* and *DFNB59* (p.G292R) performed poorly by the same measures^15^. Recently, mutated deafness genes, which were previously assumed to have a peripheral auditory function, have been proposed to elicit the gene-specific abnormalities in the central auditory region and to affect its potential network interplaying with central auditory region^16,17^. Specifically, the possibility that central auditory intrinsic deficits coexist with peripheral auditory deficits has been recapitulated by a mouse mutant model lacking both cadherin proteins (i.e. cadherin23 and cadherin15), since both proteins are required for the development of GABAergic interneurons in the auditory cortex^18^. In this regard, identification of pathogenic variants via MGT can be a crucial component in the preoperative evaluation of CI for the prognostic perspectives^15^. However, to the best of our knowledge, the clinical implications of knowing the underlying genetic etiology of a routine practice have not been clarified in postlingual deafness. Whether genetic information on post-lingual deafness–alone or in conjunction with other clinical factors– contributes to the prediction of CI outcomes may be important to acknowledge to optimize the CI outcomes, given that clinical predictors influence the brain plasticity in post-lingually deafened implantees^8,19^.

In the present study, it was observed that more than half of the post-lingual sensorineural deafness necessitating CI is related to a Mendelian genetic disease. None of the genes were particularly more prevalent than others, and many genes were found to have similar frequencies of occurrence, revealing etiologic heterogeneity. The authors evaluated the CI outcomes of these subjects as a reference to the molecular genetic etiology and clinical predictors, comparing the results of subjects with a clarified genetic aetiology with those who do not carry identified variants of known deafness genes. Thus, we suggest a differential degree of correlation between the duration of deafness and CI outcomes, depending on currently identifiable genetic aetiology.

## Results

### Clinical characteristics and demographics

Forty subjects who underwent MGT were allocated into one of the two following groups, depending on whether we could specify a definitive causative variant among the known deafness genes (https://hereditaryhearingloss.org/): the GD group, if a causative variant was identified among the known deafness genes, and the GUD group, if such identification was not possible. Clinical characteristics and demographics of each group are shown in Table 1. The deafness duration of the GD group and GUD group were 43.2 ± 7.4 months and 36 months (range from 8 to 180 months), respectively. There was no significant difference in the duration of deafness between the two groups (P = 0.469). One subject in the GD group (4.8%) and three subjects in the GUD group (15.8%) had a deafness duration of more than 10 years, revealing no statistical significance (P = 0.331). There was no significant difference in sex (P = 0.816), age at CI (P = 0.876), duration of hearing aid use (P = 0.133), the presence of inner ear anomaly (P = 0.787), and pre-CI speech evaluation scores (K-CID, P = 0.146; Spondee word, P = 0.053; PB word, P = 0.081) between the two groups. Based on the radiological evaluation, two affected subjects carrying *SLC26A4* variants and one affected subject carrying *NF2* variants in the GD group had bilateral enlarged vestibular aqueduct and bilateral vestibular schwannoma, respectively. Particularly, none of the 40 subjects included in this study had bilateral CIs, including either sequential or simultaneous implants.

**Table 1:** Demographics and clinical characteristics between GD and GUD group.

	GD (n = 21)	GUD (n = 19)	P-value
Sex (M:F)	7:14	7:12	0.816
Age at CI (years)	32.3 ± 4.1	41.5 ± 4.0	0.876
Deaf duration (months)	43.2 ± 7.4	36 [8–180]^a^	0.469
Duration of hearing aid use (months)	88.6 ± 17.5	55.7 ± 11.5	0.133
Inner ear anomaly	2 (9.5%)	0 (0.0%)	0.787
Pre-CI KCID	15.7 ± 4.1	22.3 ± 6.6	0.146
Pre-CI spondee word	13.4 ± 3.1	16.3 ± 4.4	0.053
Pre-CI PB word	13.1 ± 2.9	19.6 ± 4.3	0.081

Data are presented as mean ± standard error mean (SEM) for numeric variables if they are compatible with normal distribution on the basis of Kolmogorov-Smirnov test.

GD: genetically determined; GUD: genetically undetermined; M: male; F: female; Pre-CI: preoperative cochlear implant; KCID: Korean version of Central Institute for the Deaf; PB: phonetically balanced word test.

^a^Deaf duration of GUD group is described using median value (range) due to non-normal distribution.

### Extreme heterogeneity of the genetic aetiologies and its pathogenicity

Molecular genetic aetiology of post-lingually deafened implantees was identified in 21 (52.5%) of 40 subjects. Individualized genotype information of the GD group is summarized in Table 2. In total, 14 deafness genes were involved, which showed heterogeneity of molecular aetiology in our cohort. The most frequent causative deafness gene was *TMC1* (DFNA36) (3 cases), followed by the next tier of genes (2 cases each) (*CDH23*, *COCH*, *SLC26A4*, *TMPRSS3*, *ATP1A3*), and the genes that were detected only once (*ACTG1*, *GJB2*, *ILDR1*, *MYO7A*, *MYO15A*, *NF2*, *NLRP3* and *SERPNB6*). Moreover, any single mode of inheritance did not account for the majority of post-lingually deafened implantees; autosomal dominant (AD), autosomal recessive (AR), and de novo inheritance accounted for 33.3% (7 out of 21), 47.6% (10 out of 21), and 19.0% (4 out of 21), respectively. Whenever possible, segregation analysis was performed for all the participating family members. A missense variant of *MYO7A* gene, p.Gln752Ter, was assumed as a possible *de novo* event in SH53-118. Unfortunately, neither medical data nor DNA were available from the parents; therefore, it could not be determined whether p.Gln752Ter arose definitely a *de novo* occurrence, even though it was detected as a single heterozygote and was predicted to being pathogenic. Specifically, variants from seven genes (*ACTG1, ATP1A3, COCH, MYO7A, NF2, NLRP3*, and *TMC1*) exerted their pathogenic effect as either AD inheritance or *de novo* occurrence of the AD gene whereas, causative variants from following seven genes (*TMPRSS3, CDH23, GJB2, ILDR1, MYO15A, SERPINB6*, and *SLC26A4*) were always associated with AR inheritance for manifestation of the deafness phenotype in post-lingually deafened subjects.

**Table 2:** Individualized genotype in postlingually deafened cochlear implantees.

Subject	Gene	HGVS nucleotide: protein change	REVEL^a^	In silico computational				GERP^f^	Variant frequencies		Zygosity	Inheritance	MGT
				CADD^b^	MT^c^	SIFT^d^	PP-2^e^		KRGDB^g^ (1722 individuals)	GMAF^h^			
SB358–699	*ACTG1*	[NM_001199954.1] c.1013 C > T:p.Ser338Leu	0.883	25.6	DC	Not predicted	0.999 (D)	3.07	ND	ND	Het	AD	WES
SH191–430	*ATP1A3*	[NM_001256214] c.2491 G > A:p.Glu831Lys	0.967	26.5	DC	0.0 (D)	1.0 (D)	3.88	ND	ND	Het	de-novo AD	WES
SH222–518	*ATP1A3*	[NM_001256214] c.2491 G > A:p.Glu831Lys	0.967	26.5	DC	0.0 (D)	1.0 (D)	3.88	ND	ND	Het	de-novo AD	WES
SB116–208*	*CDH23*	[NM_022124.5] c.719 C > T:p.Pro240Leu	0.516	25.8	DC	0.001(D)	0.704 (PD)	5.19	T = 0.001455/5	T = 0.00004 (10/249236, GnomAD exome) T = 0.00009 (11/120716, ExAC) T = 0.000 (1/5008, 1000 G)	Comp het	AR	D130
		[NM_022124.5] c.5996 C > G:p.Thr1999Ser	0.086	14.01	N	Not predicted	0.0 (B)	4.14	G = 0.168899/582	G = 0.42292 (104457/246988, GnomAD_exome) G = 0.42914 (51084/119038, ExAC) G = 0.345 (1729/5008, 1000 G)			
SH62–147	*CDH23*	[NM_022124.5] c.6604 G > A:p.Asp2202Asn	0.732	18.84	DC	Not predicted	1.0 (D)	5.06	ND	A = 0.00002 (3/125568, TOPMED)	Comp het	AR	D200
		[NM_022124.5] c.5747 G > A:p.Arg1916His	0.736	25	DC	Not predicted	1.0 (D)	4.28	A = 0.001747/6	A = 0.00003 (7/237774, GnomAD_exome) A = 0.00001 (1/125568, TOPMED) A = 0.0001 (4/67492, ExAC)			
SB200–388	*COCH*	[NM_001135058.1] c.113 G > A:p.Gly38Asp	0.721	27.9	DC	0.004 (D)	0.997 (D)	5.67	ND	ND	Het	AD	TES
SH14–37	*COCH*	[NM_001135058.1] c.113 G > A:p.Gly38Asp	0.721	27.9	DC	0.004 (D)	0.997 (D)	5.67	ND	ND	Het	AD	D80
SH185–419	*GJB2*	[NM_004004.5] c.235del:p.Leu79Cysfs*3	NA	32	DC	NA	NA		del = 0.005807/20	del = 0.00036 (44/121376, ExAC) del = 0.0005 (15/30968, GnomAD) del = 0.002 (8/5008, 1000 G)	Comp het	AR	Sanger sequencing
		[NM_004004.5] c.578 T > A:p.Val193Glu	0.868	25.5	DC	0.002 (D)	0.979 (D)	5.65	ND	ND			
SH64–149	*ILDR1*	[NM_001199799.1] c.206 C > A: pPro69His	0.792	26.9	DC	0.023 (D)	1.0 (D)	5.64	ND	T = 0.00003 (7/251074, GnomAD_exome) T = 0.00006 (7/125568, TOPMED) T = 0.00004 (5/116010, ExAC)	Homo	AR	D200
SH53–118	*MYO7A*	[NM_000260.3] c.2254 C > T:p.Gln752Ter	NA	41	DC	NA	NA	5.03	ND	ND	Het	Possibly de-novo AD	D80, D200, WES
SB224–437	*MYO15A*	[NM_016239.3] c.9790 C > T:p.Gln3264Ter	NA	51	DC	NA	NA	5.61	ND	ND	Comp het	AR	TES
		[NM_016239.3] c.10263 C > G:p.Ile3421Met	0.582	23.1	DC	0.032 (D)	0.905 (D)	2.74	G = 0.000874/3	G = 0.00003 (7/249476, GnomAD_exome) G = 0.00003 (4/120692, ExAC)			
SB181–344	*NF2*	[NM_000268.3] c.932_935del: p.Arg311Lysfs*10	NA		DC	NA	NA		ND	ND	Het	AD	Sanger sequencing
SH41–90	*NLRP3*	[NM_001243133.1] c.1043 C > T: p.Thr348Met	0.776	28.8	DC	0.055 (T)	0.999 (D)	3.84	ND	ND	Het	de-novo AD	D80, D200, WES
SB114–206	*SERPINB6*	[NM_001271822.2] c.928del: p.Glu310Serfs*43	NA		DC	NA	NA	4.21	ND	ND	Compound het	AR	WES
		[NM_001271822.2] c.772-1 G > A	NA	31	DC	NA	NA	4.67	ND	T = 0.0000 (1/31404, GnomAD)			
SH100–214	*SLC26A4*	[NM_000441.2] c.919-2 A > G	NA	24.8	DC	NA	NA	5.62	G = 0.000873/3	G = 0.00036 (90/251010, GnomAD_exome) G = 0.00052 (65/125568, TOPMED) G = 0.00031 (37/121000, ExAC)	Comp het	AR	Sanger sequencing
		[NM_000441.2] c.2168 A > G:p.His723Arg	0.933	26.8	DC	0.001 (D)	1.0 (D)	5.51	G = 0.005824/20	G = 0.00012 (30/251294, GnomAD_exome) G = 0.00006 (8/125568, TOPMED) G = 0.00012 (15/121166, ExAC) G = 0.000 (2/5008, 1000 G)			
SH24–53	*SLC26A4*	[NM_000441.2] c.916dup: p.Val306Glyfs*24	NA	35	DC	NA	NA		ND	dupG = 0.00001 (3/251278, GnomAD_exome) dupG = 0.00002 (2/121236, ExAC)	Comp het	AR	Sanger sequencing
		[NM_000441.2] c.2168 A > G:p.His723Arg	0.933	26.8	DC	0.001 (D)	1.0 (D)	5.51	G = 0.005824/20	G = 0.00012 (30/251294, GnomAD_exome) G = 0.00006 (8/125568, TOPMED) G = 0.00012 (15/121166, ExAC) G = 0.000 (2/5008, 1000 G)			
SB144–238	*TMC1*	[NM_138691.2] c.1714G > A:p.Asp572Asn	0.465	29.7	DC	0.122 (T)	0.999 (D)	6.16	ND	ND	Het	AD	D80, WES
SB144–239	*TMC1*	[NM_138691.2] c.1714G > A:p.Asp572Asn	0.465	29.7	DC	0.122 (T)	0.999 (D)	6.16	ND	ND	Het	AD	D80, WES
SB279–550	*TMC1*	[NM_138691.2] c.1714G > A:p.Asp572Asn	0.465	29.7	DC	0.122 (T)	0.999 (D)	6.16	ND	ND	Het	AD	WES
SH174–387	*TMPRSS3*	[NM_024022.2] c.346 G > A:p.Val116Met	0.695	28.1	DC	0.026 (D)	1.0 (D)	4.94	ND	T = 0.00005 (13/251420, GnomAD_exome) T = 0.00003 (4/125568, TOPMED) T = 0.00006 (7/121402, ExAC)	Homo	AR	D130
SH51–112	*TMPRSS3*	[NM_024022.2] c.916 G > A:p.Ala306Thr	0.851	34	DC	0.002 (D)	0.999 (D)	4.8	T = 0.001164/4	T = 0.00014 (36/249060, GnomAD_exome) T = 0.00012 (15/125568, TOPMED) T = 0.00017 (21/121412, ExAC) T = 0.000 (1/5008, 1000 G)	Comp het	AR	D80, D200, WES
		[NM_024022.2] c.325 C > T:p.Arg109Trp	0.767	28.4	DC	0.0 (D)	1.0 (D)	3.99	A = 0.000588/2	A = 0.00013 (33/251350, GnomAD_exome) A = 0.00010 (13/125568, TOPMED) A = 0.00010 (12/121392, ExAC)			

HGVS, human genome variation society; Homo, homozygosity; Comp Het, compound heterozygosity; Het, heterozygosity; DC, disease causing; D, deleterious; N, neutral; B, benign; NA, not available; ND, not determined; WES: whole exome sequencing; TES: targeted exome sequencing; D80, D130, and D200: Deafness panel comprising 80 genes, 130 genes and 200 genes, respectively.

^a^Rare Exome Variant Ensemble Learner (REVEL; https://sites.google.com/site/revelgenomics/about).

^b^Combined Annotation Dependent Depletion (CADD; https://cadd.gs.washington.edu/).

^c^Mutation taster (http://www.mutationtaster.org/).

^d^Sorting Intolerant from Tolerant (SIFT; http://sift.jcvi.org/).

^e^PolyPhen-2 (PP2) prediction score (HumanVar), ranges from 0 to 1 (0 = benign, 1 = probably damaging http://genetics.bwh.harvard.edu/pph2/).

^f^Genomic Evolutionary Rate Profiling (GERP++; http://genome.ucsc.edu/).

^g^Korean reference genomic database (KRGDB; http://coda.nih.go.kr/coda/KRGDB/index.jsp).

^h^Global minor allele frequency.

Exome Aggregation Consortium databases (ExAC; http://exac.broadinstitute.org/).

Genome Aggregation Database (GnomAD; https://gnomad.broadinstitute.org/),.

NHLBI Trans-Omics for Precision Medicine (TOPMed; https://bravo.sph.umich.edu/freeze3a/hg19/),.

1000 Genomes Project (1000 G; http://grch37.ensembl.org/Homo_sapiens/Info/Index).

*The causal relationship between *CDH23* alteration and Postlingual SNHL in SB116 was previously described (Kim *et al*.)^8^.

As documented in previous studies^13^, we further divided the subjects with a definite molecular genetic diagnosis into two groups based on the primary expression site of the gene; ‘membranous labyrinth (ML)’ group and ‘spiral ganglion neurons (SGN)’ group. The ML-related genes were exclusively expressed in the inner ear, while the SGN-related genes were expressed abundantly in SGN and brainstem auditory nuclei. The ML group included subjects with causative variants of *ACTG1, ATP1A3, CDH23, GJB2, ILDR1, MYO7A, MYO15A, NLRP3, SERPINB6, SLC26A4, and TMC1*, while the SGN group included subjects with causative variants of *COCH*, *TMPRSS3*, and *NF2*, which are expressed abundantly in postsynaptic sites or SGNs.

We provide here compelling evidence supporting that the variants observed from GD group are pathogenic **(**Table 2**)**. The variants demonstrated a significantly lower detection frequency with a cut-off threshold for AR (≤0.00007 [0.0007%]) or AD (≤0.00002 [0.0002%]) inheritance in the Korean Reference Genome Database (KRGDB, 1722 individual) or Global Minor Allele Frequency (GMAF). In addition, the variants were consistently predicted to be pathogenic, as ‘damaging’ or ‘probably damaging’ by at least some, if not all, comprehensive *in silico* computational analyses. Moreover, the residue of variants to be examined was evolutionarily well-conserved among several species, which was supported by a GERP++ score of higher than 3 except p.Ile3421Met of the *MYO15A* gene.

In SB 116–208, p.Pro240Leu allele of *CDH23*, a founder effect in Korea, was known to potentially lead to adult-onset postlingual SNHL, when it is in a *trans* configuration with a milder pathogenic potential, as revealed by our previous study^20^. Thus, some of the frequent neighboring single nucleotide polymorphisms (SNPs), which was identified in a *trans* configuration with the p.Pro240Leu allele, have been suggested to exert an epistatic effect, which could contribute to post-lingual deafness.

### Postoperative Outcomes between GD and GUD group

**Figure 1:**
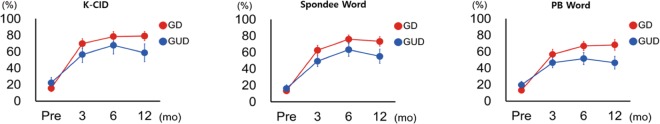
Comparison of longitudinal change of speech perception scores between the GD and GUD groups. There was no significant difference in speech perception scores between two groups at any time point, from baseline to 12 months (as seen through Supplement Fig. 1). The speech perception scores significantly increased within 3 months after cochlear implantation, and subsequently reached a plateau at the 6-month postoperative evaluation, regardless of the group. The degree of reflection of the speech perception scores at 3 months was more than 0.8 in both the GD and GUD groups. The speech perception score at 12 months was almost the same as the 6-month score.

All subjects enrolled in the current study participated in speech evaluation. Speech perception performance was assessed by the Korean version of the Central Institute for the Deaf (K-CID) and Spondee and phonetically balanced (PB) word test without visual cues at pre- and post-operative 3, 6, and 12 months^21^. As shown in Fig. 1, the degree of reflection of 1-year results for the K-CID, Spondee, and PB scores at 3 months postoperatively was 0.83 (0.14–1.24), 0.80 (0.23–1.33), and 0.83 (0.42–1.13) in the GD group, respectively, and 0.97 (0.04–3.00), 0.85(0.07–3.33), and 0.94 (0.34–4.55) in the GUD group, respectively. The degree of reflection of 1-year result by 3 month’s speech perception scores was not significantly different  between the two groups (P = 0.297, 0.388, and 0.235). The degree of reflection refers to the percentage of improvement of speech perception scores measured at a specific time point as compared with that obtained at 1- year follow up.

**Figure 2:**
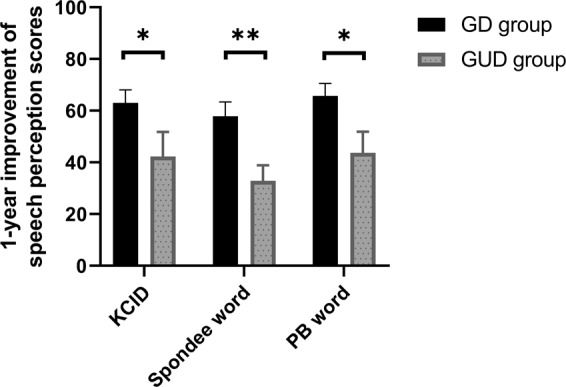
Comparison of the improvement during postoperative 1-year follow-up. The improvement refers to the difference in speech perception scores between preoperative and 1-year postoperative timepoints. A significant improvement during the postoperative 1-year follow-up was found in the GD group compared to that in the GUD group (by independent t-test). *<0.05, **<0.005.

Longitudinal data on speech perception scores showed that there were no significant differences of the K-CID, Spondee, and PB word scores at time point of pre- and post-operative 3, 6, and 12 months between these two groups (Supplement Fig. 1). However, the GD group exhibited longitudinal changes with a significant improvement from preoperative evaluation to 12 months postoperative in K-CID, Spondee, and PB word scores (63.1 ± 5.0 vs. 42.3 ± 9.5, P = 0.048, 57.8 ± 5.5 vs. 32.9 ± 6.0, P = 0.005, and 65.7 ± 4.9 vs. 43.7 ± 8.2, P < 0.001, respectively), compared to the GUD group (Fig. 2). The presence of identifiable causative variants among the known deafness genes was independently associated with significantly better CI outcomes during the 1-year follow-up period, even after adjusting for the clinical factors including duration of deafness, age at CI, and duration of hearing aid use (K-CID: β:29.9, SE:9.3, and P = 0.006; Spondee, β:29.3, SE:8.8, and P = 0.003; PB, β:32.247, SE:7.3, and P < 0.001) **(**Table 3**)**.

**Table 3:** Factors associated with 1-year improvement of speech perception scores.

	Estimate (*β)*	Standardized	Standard error	P-value
**Univariable regression**				
Presence of genetic aetiology	K-CID:27.973	K-CID:0.487	K-CID:9.325	K-CID:0.006*
	Spondee:29.322	Spondee:0.538	Spondee:8.831	Spondee:0.003*
	PB:32.247	PB:0.673	PB:7.250	PB: <0.001*
Duration of deafness	K-CID:−0.100	K-CID:−0.342	K-CID:0.051	K-CID:0.06*
	Spondee:−0.088	Spondee:−0.315	Spondee:0.051	Spondee:0.096
	PB:0.029	PB:−0.069	PB:0.048	PB:0.161
Age at CI	K-CID:−0.067	K-CID:−0.044	K-CID:0.281	K-CID:0.812
	Spondee:-0.088	Spondee:-0.062	Spondee:0.275	Spondee:0.750
	PB:−0.114	PB:−0.085	PB:0.257	PB:0.661
Duration of hearing aids use	K-CID:0.056	K-CID:0.193	K-CID:0.053	K-CID:0.297
	Spondee:0.031	Spondee:0.103	Spondee:0.057	Spondee:0.595
	PB:0.029	PB:0.105	PB:0.053	PB:0.589
**Multivariable regression**				
Presence of genetic aetiology	K-CID:24.126	K-CID:0.420	K-CID:9.792	K-CID:0.020*
	Spondee:26.556	Spondee:0.488	Spondee:9.273	Spondee:0.008*
	PB:33.174	PB:0.652	PB:7.721	PB:<0.001*

CI: cochlear implantation; K-CID: Korean version of Central Institute for the Deaf; PB: phonetically balanced word test; *statistically significance.

### Postoperative outcomes of GD group according to the known clinical variables and gene expression site

**Figure 3:**
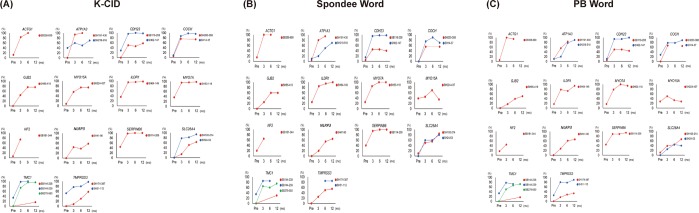
Longitudinal changes in speech perception scores from preoperative to 1-year postoperative timepoints according to the individualized deafness gene in the GD group. (**A**) K-CID, (**B**) Spondee word, (**C**) PB word.

**Figure 4:**
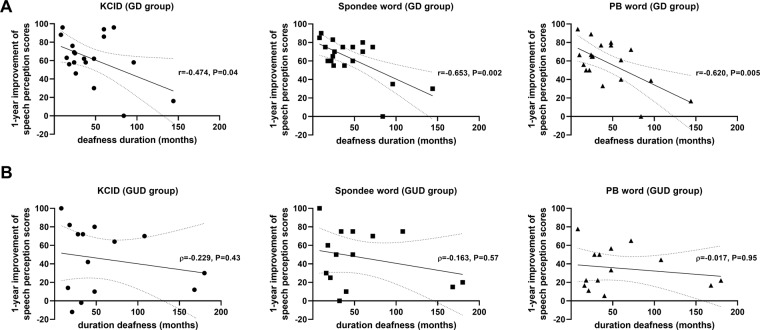
Correlation analyses of the duration of deafness and improvement in speech perception scores according to the presence of identifiable genetic etiology. (**A**) Using Pearson correlation analyses, the duration of deafness was found to be inversely correlated with 1-year improvement in K-CID (r = −0.474, P = 0.04), Spondee word (r = −0.653, P = 0.002), and PB word (r = −0.620, P = 0.005) test scores in the GD group. The dotted line indicates statistical significance. The grey color indicates the 95% confidence interval. (**B**) Using Spearman correlation analyses, the duration of deafness was found not to be inversely correlated with 1-year improvement of K-CID (ρ = −0.229, P = 0.43), Spondee word (ρ = −0.163, P = 0.57), and PB word (ρ = −0.017, P = 0.96) scores in the GUD group. The dotted line indicates the 95% confidence interval.

Individual data regarding serial scores of K-CID, Spondee, and PB word in the GD group are shown in Fig. 3. In particular, among the three patients carrying the same *TMC1* variant (p.Asp572Asn), SB144-238 with deafness duration of approximately 20 years demonstrated the K-CID, spondee, and PB word scores of 16, 30, and 16.66, respectively at 1-year evaluation. On the other hand, the remaining two subjects (SB144-239 and SB279-550) with a deafness duration of less than 10 years exhibited excellent results with a K-CID score of nearly 100% at postoperative 1-year, suggesting tight inverse correlation between deafness duration and speech outcome. Supporting this, Pearson correlation analyses revealed that the duration of deafness was inversely correlated with the 1-year improvement of speech perception scores in the GD group (K-CID: r = −0.474, P = 0.04; Spondee word: r = −0.653, P = 0.002; PB word: r = −0.620, P = 0.005) **(**Fig. 4A**)**. In contrast with the GD group, the duration of deafness was not found to be inversely correlated with the 1-year improvement of speech perception scores in the GUD group via Spearman correlation analyses K-CID (ρ = −0.229, P = 0.43), Spondee word (ρ = −0.163, P = 0.57), and PB word (ρ = −0.017, P = 0.96) **(**Fig. 4B**)**.

**Figure 5:**
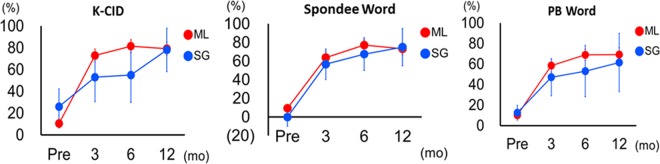
Comparison of longitudinal change in speech evaluation scores between the ML and SGN groups. The speech perception scores were seemingly higher in the ML group than in the SGN group at postoperative 3 and 6 months, but the difference did not reach a level of significance (Mann-Whitney U-test). Nonetheless, the speech perception scores in the SGN group eventually converged with those in the ML group at 1-year evaluation. Additionally, there was no significant difference in the improvement during postoperative 1-year follow-up between ML and SGN groups.

Among the GD group, the speech perception scores were seemingly higher in the ML group than in the SGN group at both 3 and 6 months postoperatively; however, the difference did not reach a level of significance due to a low number of SGN-related cases (N = 5). As illustrated in Fig. 5, the speech perception scores in the SGN group tend to gradually catch up with that of the ML group, leading to a similar auditory performance at 1-year evaluation between the two groups.

## Discussion

This study was performed to explore the CI outcomes of postlingually deafened cochlear implantees, focusing on their molecular genetic aetiology and its potential predictive capability of the CI outcome. Our comprehensive MGT protocol, including exome sequencing, enabled the enhancement of the detection of causative variants among the known deafness genes, identifying the genetic aetiology from approximately 50% in our cohort. Notably, the presence of identifiable causative variants among the known deafness genes was statistically proven to yield better CI outcomes than those without identifiable variants; however, the genetic etiology alone may not be sufficient in predicting the CI outcome, given a considerable variation among subjects with the same genotype. The duration of deafness is negatively associated with the CI outcomes, especially in subjects with identified causative variants among known deafness genes, however, not in those who remain undiagnosed even after rigorous, comprehensive MGT protocol in our cohort. Based on this, we suggest a differential correlation between the duration of deafness and CI outcomes, depending on the presence of identifiable genetic aetiology under the current MGT protocol.

We analyzed the CI outcomes focusing on whether a causative gene was revealed or not, and we also examined the outcome in accordance with the gene expression site in postlingually deafened cochlear implantees. Interestingly, molecular etiologic heterogeneity involving14 deafness genes in 21 subjects (21/40, 52.5%) was noted in this study. As shown by us previously, the proportion of *GJB2* and *SLC26A4* variants in prelingually deafened implantees accounted for up to 38% of the causes, indicating genetic preponderance^11,14,22^. Moreover, AD and AR inheritance accounted for approximately half of each in the present study, which was also different from prelingual HL where AR inheritance was predominant. Our results are in line with previous reports, showing heterogenous genetic backgrounds in postlingually deafened cochlear implantees^13,14^.

Given the nature of extreme genetic heterogeneity in postlingual deafness, genetic screening without exome sequencing may be insufficient for identification of pathogenic variants^13,14^. In this study, exome sequencing was performed for those without the phenotypic markers, and those carrying no variant in the corresponding deafness gene were screened by deafness panel sequencing (TES-129) and phenotype-driven Sanger sequencing. Notably, the causative variants have been identified by exome sequencing in the present study, in 11 out of 21 subjects. Similarly, some of the genetic studies still presented a risk of insufficient capturing or coverage of the targeted panel sequencing^23,24^. Considering that a comprehension of genetic etiologies has been implicated in selecting candidates and CI prognosis^12^, MGT including whole-exome sequencing should be implemented as a part of preoperative CI evaluation if the causative gene is not found by directive Sanger or panel sequencing especially in postlingually deafened implantees.

The current study observed that those in the group with identified causative variants among the known deafness genes had significantly better CI outcomes than those in the undiagnosed group, suggesting a potential relationship between genotype and functional outcome. Furthermore, the multivariable regression analyses indicated that the presence of identifiable causative variants among the known deafness genes was independently associated with better CI outcomes during the 1-year follow-up period even after adjusting clinical confounders. The multivariable regression analyses generally accept the common rule of thumb that the ratio of inclusion of observations to predictors be at least 10:1; therefore, in this study, regression analyses using the 4 aforementioned variables in 40 subjects were considered valid^25^. According to a previously suggested classical hypothesis, which simply postulated the prediction of CI outcomes based on SGN health^13,14^, better CI outcome in the GD group compared with the GUD group may be attributed to the dominant composition of ML-related deafness genes (11 out of 14 deafness genes), which may pose a relatively weaker risk of damage on the SGN health, if mutated. Further, even subjects with specific SGN-related deafness genes documented by our MGT results, including *COCH*^26–30^, *TMPRSS3*^31–35^, and *NF2*^36,37^, were reported to attain some extent of audiological benefit from CI. Consistent with this, our results demonstrated that speech perception performances at 1-year evaluation were not significantly different between the ML and SGN groups. Based on MR imaging, it is assumed that cochlear aperture obstruction and subsequent elevated protein within the inner ear may play a critical role in deteriorating hearing loss in subjects with *NF2* variants^38^. In line with our case with *NF2*, a previous study demonstrated that approximately 70% of *NF2* subjects have shown to achieve open-set speech discrimination with an intact cochlear nerve^37^. Meanwhile, clinical factors, such as duration of hearing loss and hearing status in the contralateral ear, were known to affect the CI outcomes in subject with *NF2* variants^36^. Furthermore, cochlear aperture obstruction, to some degree, which is correlated with the deterioration of cochlear nerve axonal transport might also be related to the variable CI outcomes^38^. Recently, one study suggested that the genotype-derived genetic severity score may predict clinical phenotypes in individual with *NF2* variants; however, the exact molecular mechanism remains unknown with respect to how *NF2* variants or their encoded proteins work in the inner ear^39^.

Based on individual analysis according to genotype, genetic aetiology alone does not likely suffice for the prediction of CI outcomes. A recent study revealed that short-term deafness (<10 years), younger age at CI, and long-term hearing aid use were key factors toward good CI outcomes in postlingual deafness^9^. Similarly, we also observed that evident differences of speech perception scores were found in GD group according to the duration of deafness (cut-off value: 10 years) **(**Supplement Fig. 2**)**. Typically, brain plasticity is one of the important factors to determine the auditory development for implantees, including postlingually deafened subjects with identifiable genetic aetiology. According to individualized genotype in the GD group, clinical variables seem to yield different CI outcomes, with the same genotype. For example, *TMC1*, which encodes a component of the mechano-transduction channel in hair cells, was known to markedly improve verbal communication after CI^40^. However, subject SB144-238 (F/54) with 12 years of deafness in this study showed a poor CI outcome throughout the follow-up period, compared with SB144-239 and SB279-550 whose deafness duration was shorter than 10 years but with the same genotype. The longer duration of deafness is likely to cause cross-modal reorganization that correlates with poor CI outcomes, whereby cortical regions of the deprived modality become vulnerable to the recruitment by the other remaining intact sensory modalities^41^. Age at implantation could also make a difference even with the same genetic aetiology and similar deafness duration. For *CDH23* variants, younger implantee (SH62-147) showed outstanding performance compared with an older implantee (SB116-218), who underwent CI at 72 years, even though their deafness duration was not significantly different. Thus, the age at CI may play an important role as a determinant for CI outcome when considering a decrease in the spiral ganglion cell population due to aging, leading to fewer neurons available for stimulation^42^. Also, a decoding of the input provided by the CI and subsequent auditory processing were based on top-down cognitive processing^43^, suggesting healthy cognitive resource is being an imperative prerequisite for desirable CI outcome. It appears that cognitive resources necessary for substantial benefit from CI may also be inversely associated with age^43^. Nonetheless, our interpretation may be limited because of the comparison among individual subjects instead of between groups. Further comparative studies would confer the additional support with respect to the need for consideration of clinical variables, even if the genetic etiology-based pathophysiological mechanism and its prognosis are revealed.

In this sense, the identification of molecular genetic aetiology in our current study could significantly impact our clinical practice. Specifically, we observed a different degree of correlation between the duration of deafness and an improvement in auditory performance, depending on the presence of identifiable genetic aetiology, given the lack of relationship of the outcome with duration of deafness in the GUD group. Using Spearman correlation analyses, the duration of deafness was found not to be inversely correlated with 1-year improvement of K-CID (ρ = −0.229, P = 0.43), Spondee word (ρ = −0.163, P = 0.57), and PB word (ρ = −0.017, P = 0.96) in the GUD group. On the other hand, a significant inverse correlation between the duration of deafness and 1-year improvement of speech perception scores was found exclusively in the GD group. However, there still remains a tendency, albeit not statistically significant, that GUD subjects with longer deafness duration show poorer outcome than those with short deafness duration. Therefore, it would be fair to say that influence of long deafness duration on CI outcome was more pronounced in the GD group rather than in the GUD group, not to say that early CI is pointless in the GUD group. Our results might indicate a new implication of genetic testing on proposing a time window for good CI outcomes with the current MGT protocol. In other words, subjects with identifiable genetic aetiology could benefit from early CI to a greater extent than those with unidentifiable genetic aetiology, which provides a rationale for early CI and avoidance of unnecessary struggle with hearing aids in the GD group.

Much less tight correlation was observed between the long deafness duration and the poor outcome in the GUD group compared with the GD group. There could be a non-genetic aetiology, preferentially inducing cortical deficits concomitantly with a peripheral organ dysfunction in the GUD group. These intrinsic cortical deficits would potentially render the cortical issues and plasticity, which was already intrinsically damaged, to be less sensitive to clinical variables, such as deafness duration. This phenomenon would not be limited to non-genetic etiology. Based on the gene regulatory network, recent studies suggest a possibility of concomitant and widespread expression of deafness genes or their encoded proteins beyond the cochlea^16^. Moreover, some authors proposed that some specific deafness genes may contribute to the development in both the central- and peripheral auditory systems^17,18^. An animal model of congenital deafness caused by variants of *PCDH15*, encoding cadherin-related transmembrane proteins, has shown to hinder the auditory cortex interneuron development, and therefore, coexist with the peripheral deficits^18^. This finding can be translated into a human study exhibiting unusual speech recognition difficulties after CI in subjects with *PCDH15* variants^15^. In other words, at least a proportion of subjects in the GUD group may have novel genetic variants which have not yet been identified and are predisposed to affect both peripheral and central auditory systems, like the *PCDH15* variants or mainly in the central ones. Thus, a possible involvement of the unknown deafness genes related to hidden intrinsic cortical deficits in the GUD group may explain our results demonstrating relatively poor CI outcomes in the GUD group compared with the GD group^18^. The GD group did not include *PCDH15* variants in our study.

In this study, the speech perception scores significantly increased within 3 months after CI and subsequently reached a plateau at 6-month postoperative evaluation, regardless of the group. It can be recapitulated by the degree of reflection of speech perception scores at 3months postoperatively that demonstrates more than 0.8 in both the GD and GUD groups. Interestingly, the scores in the GUD group appear to show a slight decrease from 6 months to 12 months (Fig. 1), but the mean difference of spondee word scores between 6 months and 12 months was 2.8 (SEM: 11.78). Considering the significant standard deviation, it is expected that the difference between 6 months and 12 months would decrease as the number of cohorts increases. Meanwhile, the Spondee word score in the GD group also showed a slight decrease at 12 months compared to that at 6 months, which is in contrast to the changes in K-CID and PB word scores that exhibited a slight increase. If these changes are driven by the aging effects characterized by a decreasing number of neurons for electrical stimulation, there would be no discrepancy among speech perception test results in the same group. Additionally, new regression analysis, including preoperative scores, showed that preoperative scores did not significantly contribute to the improvement of speech perception scores. Given this, it is least likely that slightly better preoperative scores in the GUD group exert a significant effect on the difference in speech perception score improvement between two groups.

There are some limitations in this study that should be addressed in future studies. First, the present study was limited by a relatively small number of study subjects, potentially leading to misinterpretation. Second, the longitudinal changes in language development for more than 12 months were not available. Thus, a large cohort study with longitudinal follow-up might be warranted to validate our observations. Lastly, based on our results, the presence of genetic aetiology theoretically predicts the CI outcomes of at least up to 1-year follow up. The mapping strategy of cohorts included in this study was carried out in the same manner as specified, but the time for cochlear implant use is not specified in detail. Additionally, the age-appropriate cognitive evaluation was not conducted even though a psychiatric examination performed as part of the CI work-up did not show any cognitive impairment that might precipitate dementia and severe psychiatric diseases in these cohorts. Nevertheless, our results support the notion that comprehensive, preoperative, molecular genetic evaluation should be indicated in postlingually deafened CI candidates.

## Conclusion

A meaningful genetic contribution is observed from postlingually deafened CI implantees, when deafness is limited to pure sensorineural origin. In addition to this unexpectedly high genetic contribution, extremely heterogenous genetic aetiology is also noted, requiring comprehensive genetic testing for this group. The presence of pathogenic variants among the known deafness genes was statistically proven to elicit more significant outcomes than the absence of identifiable variants. Further, there seems to be a much tighter correlation between the long duration of deafness and poor auditory performance in GD group than in GUD group, which provides a strong rationale for recommendation of early CI especially for those with a proven genetic aetiology.

## Methods

### Participants

Forty-eight post-lingual deafened subjects who consented to MGT and underwent CI between January 2010 and March 2017 at Seoul National University Hospital and Seoul National University Bundang Hospital were eligible to participate in this study. Subjects with the following conditions were excluded from this study for a legitimate CI outcome comparison: 1) severe chronic otitis media or otosclerosis that provides a significant conductive component 2) history of explantation or reimplantation, and 3) implantation by unproven CI device (e.g., NUVOC1, currently discontinued products).

As a result, 40 subjects were enrolled eventually. CI surgery for our subjects in this study was performed exclusively by two experienced surgeons (B.Y.C. & S.H.O). Of the 40 subjects, 22 subjects had CI devices from Cochlear Corp. (Lane Cove, New South Wales, Australia) implanted, along with various types of electrodes and speech processors. Seventeen were implanted with devices from MED-EL (Innsbruck, Austria), and one with a device from Advanced Bionics, Corp. (Sylmar, CA, USA). The study was approved by the Institutional Review Board of the Clinical Research Institute at Seoul National University Bundang Hospital and conducted in accordance with the Declaration of Helsinki (IRB-B-1708-412-128); the requirement for informed patient consent was waived.

### Measurement of speech perception performance

For preoperative speech evaluation, the test scores were obtained when the subjects were using their hearing aids. The K-CID test, designed to assess understanding of speech cognition in everyday conversational situations, was scored based on the percentage of correctly identified words. The speech perception performance was evaluated by word-recognition tasks using spondees and phonetically balanced (PB) words at 70 dB SPL in an audio-only condition.

### Molecular genetic testing (MGT)

MGT was conducted before CI for all the subjects. Identification of genetic aetiologies was based on the following process: (1) Direct Sanger sequencing in subjects with a characteristic phenotypic marker, such as enlarged vestibular aqueduct, and (2) deafness panel sequencing (TES-129) or additional exome sequencing in subjects without phenotypic markers or without variants in the corresponding deafness gene^44,45^. The targeted exome sequencing (Otogenetics, Norcross, GA, USA), which was observed by the NimbleGen Sequence Catcher (Roche NimbleGen Inc., Madison, WI, USA), was tested against 134 known deafness genes. The readings were compared to the UCSC hg19 reference genome, and non-synonymous SNPs were filtered with a depth = 40; dbSNP138 was filtered out, except for the flagged SNP.

For subjects who did not carry convincing variants in the deafness panel or did not display any specific clinical phenotypic marker, exome sequencing was performed, and bioinformatics analyses were conducted, as previously described^24,46^. In detail, the obtained readings were mapped onto the UCSC hg19 reference genome assembly using the Lasergene 14 software package (DNASTAR, Madison, WI, USA) and rare single-nucleotide variations (SNVs), indels, or splice-site variants were selected via a multiple filtering process^45^. As an initial step of basic filtering, non-synonymous SNPs with a quality score greater than 30 and a read depth of more than 20 were selected. The previously known disease-causing SNPs, or SNPs with GMAF ≤ 0.002, as well as with allele frequency <0.005 in ethnicity matched controls consisting of 1,722 Korean individuals (KRGDB), were chosen. Subsequently, the presence of variants was confirmed through Sanger sequencing, and a segregation study was performed. An in-silico study using SIFT (http://sift.jcvi.org/) and PolyPhen2 (http://genetics.bwh.harvard.edu/pph2/) was conducted to predict the pathogenic potential of each detected variant. Additionally, the GERP++ score from the UCSC Genome Browser (http://genome.ucsc.edu/) was utilized to estimate the evolutionary conservation of the amino acid sequences.

### Statistical analysis

All the analyses employed and illustrated used the GraphPad Prism version 8.0.0 for Windows, GraphPad Software, San Diego, California USA (www.graphpad.com), except for regression analyses. Results are presented as mean ± standard error mean (SEM). Independent t-test, Mann-Whitney U test, overall exact chi-square tests, and Fisher exact test were used as appropriate to compare demographic and clinical characteristics. Between the GD and GUD groups, the improvement in auditory performance between pre- and post-operative 12 months was compared using an independent t-test. According to normal distribution of data, Pearson and Spearman correlation analysis was employed to analyse the relationship between the CI outcomes (improvement of speech perception scores between pre- and post-operative 12 months) and the duration of deafness, as appropriate. Whenever available, uni- and multivariable regression analyses were performed to simultaneously assess the relative influence of CI outcomes and associated variables, including the presence of genetic aetiology, duration of deafness, age at CI, and duration of hearing aid use. The regression analyses were performed using the software package R (version 3.3.2, R Foundation for Statistical Computing, Vienna, Austria). P-values of <0.05 were considered to indicate statistical significance.

## Supplementary information

Supplementary information.

Supplementary information 2.

## Data Availability

Data for all submitted results is available.
