# Supplementary material for: The molecular etiology of deafness and auditory performance in the postlingually deafened cochlear implantees
Source: Sci Rep. 2020 Apr 1;10:5768. doi: 10.1038/s41598-020-62647-y (PMC7113281; doi:10.1038/s41598-020-62647-y)
Supplement: Supplementary file 1 — Supplementary information. [file 41598_2020_62647_MOESM1_ESM.pdf]

The molecular etiology of deafness and auditory performance in the postlingually deafened cochlear implantees

Sang-Yeon Lee[1§], Ye Ji Shim[2§], Jin-Hee Han[1], Jae-Jin Song[1], Ja-Won Koo[1], Seung Ha Oh[3], Seungmin Lee[1], Doo-Yi Oh[1], Byung Yoon Choi[1*]

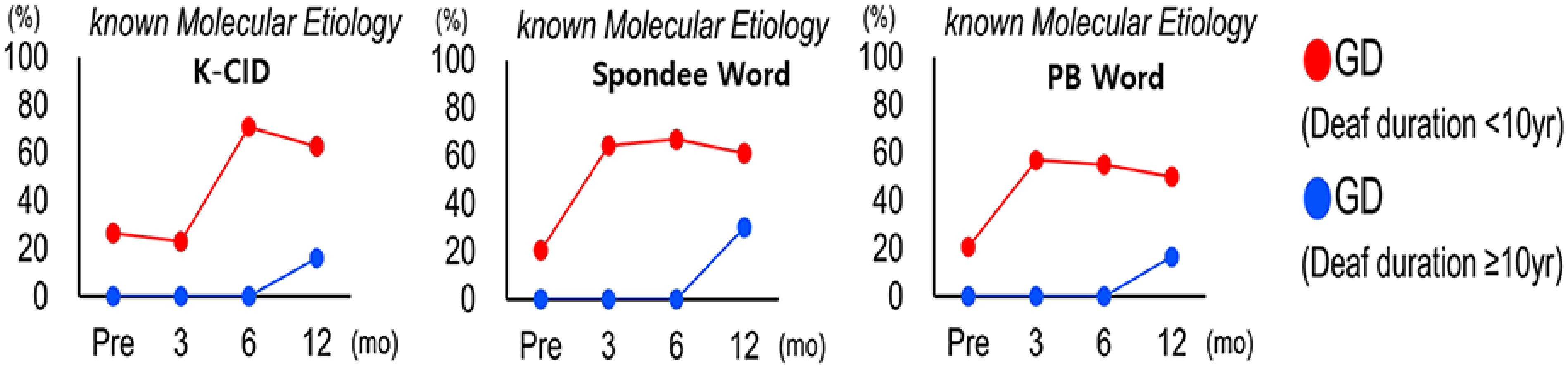

known Molecular Etiology
K-CID
Spondee Word
PB Word
(%)
100
80
60
40
20
0
Pre
3
6
12
(mo)
GD
(Deaf duration <10yr)
GD
(Deaf duration ≥10yr)
