# Supplementary figures and images for: The molecular etiology of deafness and auditory performance in the postlingually deafened cochlear implantees
Source: Sci Rep. 2020 Apr 1;10:5768. doi: 10.1038/s41598-020-62647-y (PMC7113281; doi:10.1038/s41598-020-62647-y)

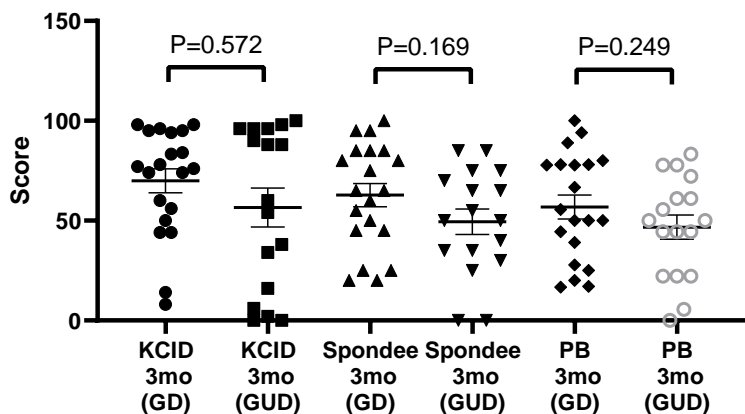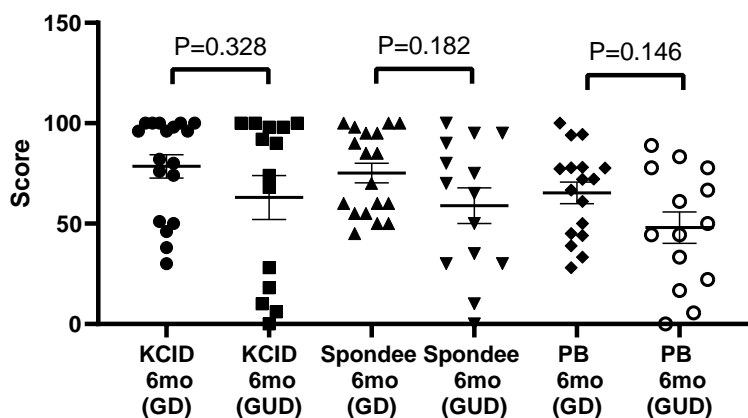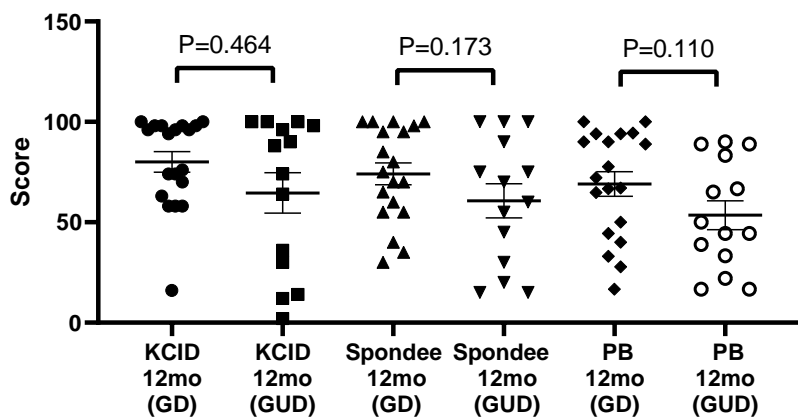

Supplement: Supplementary file 2 — Supplementary information 2. [file 41598_2020_62647_MOESM2_ESM.pdf]
